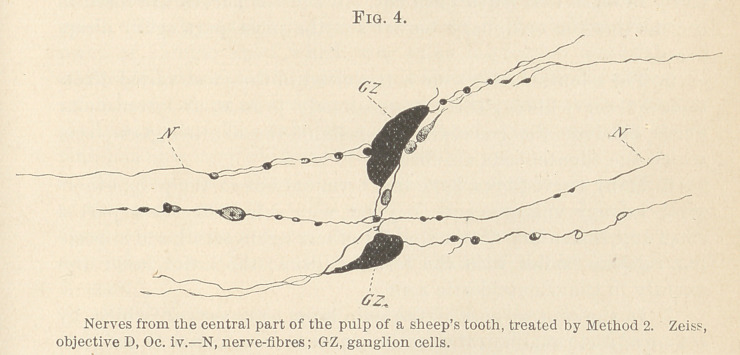# Investigations of the Nerves of the Pulp by the Methylene-Blue Method

**Published:** 1897-04

**Authors:** Michael Morgenstern

**Affiliations:** Germany


### Abstracts and Translations.

### INVESTIGATIONS OF THE NERVES OF THE PULP BY THE METHYLENE-BLUE METHOD.[1]

BY MICHAEL MORGENSTERN, GERMANY.

By this method I never succeeded in coloring all the nerves of the pulp blue, and for this reason I used it principally to establish certain special points concerning which the employment of other methods had left me in doubt. Among these were the innervation of the blood-vessels, the relation of the axis-cylinder to various cellular components, the nerve-endings, and the structure of the nerve plexus below and within the odontoblastic zone.

The innervation of the blood-vessels in the pulp is enormous; all the arteries, even the smallest arterioles, exhibited an extremely fine and elaborate plexus. In the larger blood-vessels this plexus shows a very regular arrangement of its fibres, one part of

[1] Abstract from a paper in the Deutsche Monatschrift für Zahnheilkunde, September, 1896.

which runs parallel with the blood-vessels and another at right angles to them; in this way each nucleus is made to rest, as it were, in a net-work of nerves. One or more axis-cylinders are frequently found with the finest capillaries, not uncommonly embracing these in spirals. This was especially easy to establish in the crown pulp of a calf, which sends out a large number of tufted ramifications into the dentine, each tuft in turn consisting of an indefinite num-

Fig. 1.

Nerve-endings from the crest of the crown pulp of a new-born calf, treated by Method 2. Zeiss $\frac{1}{12}$, oil immersion, Oc. ii.—N, nerve-fibres; NE, disk-like terminal bodies of nerves; C, capillary of pulp, by which the nerve N ascends; Rz, Ranvier cells; NF, small terminal nerve-fibre; EA, terminal enlargement of the nerve.

ber of projecting parts; each of these parts contains an acute-angled capillary loop, which projects in the form of a small peg, and about which the dentine cells are arranged very much as the cells are grouped about the blood-vessels of the central or axial part in the dentine germ of young pikes, from which vaso-dentine is formed. Now, among these partly-formed cells there are some in which the capillaries are spirally surrounded by axis-cylinders, while nerve-endings can frequently be recognized between them. The axis-cylinder then separates itself from its capillary, rises generally rather higher than the latter, and then turns in towards the narrow intermediate space mentioned above, where it terminates in a small ovally rounded body; this often hangs from it like a berry from its stem. (Fig. 1.) As I have also frequently observed that these axis-cylinders terminate similarly at these points in a small elongated body, I believe I must assume that the two bodies are only two

FIG. 2.

Nerves from the pulp of a calf's tooth, colored with methylene blue according to Bethe. Zeiss $\frac{1}{12}$, oil immersion, Oc. ii.—Rz, contractions between Ranvier cells; REF, brush-like ending of a nerve.

different aspects of one and the same formation, which accordingly must have the form of a disk. I note here, parenthetically, that I have found analogous formations in dentine that had been treated by the corrosive sublimate method (Golgi), and had afterwards been colored with hæmotoxylin; but in such cases these disks have more commonly the form of a rhombus with rounded angles.

Most of the nerves show throughout their more or less considerable extent an undulating, spiral, or zigzag course; the larger fibres have Ranvier cells at fairly regular intervals, the finer ones have few cells or even none at all, although they have many knobby enlargements, which appear in the finest fibres only as points. (Fig. 2.)

It is difficult to determine nerve-endings in the pulp with certainty, because one can never assert with absolute assurance that where a fibre appears to terminate, it does not in reality continue in some other direction that lies outside of our field of observation. Nevertheless, I believe that the great attenuation of many fibres warrants the conclusion that they terminate as free fibres, especially when they divide, like a brush, into a number of very fine threads. (Fig. 2, REF.) It was possible, however, to establish more positively the fact that many axis-cylinders terminate in small, knobby enlargements, beyond which for the most part a very fine, short thread extends. (Fig. 1, NF.)

Between and below the odontoblasts the nerves frequently terminated in rounded cellular bodies, which could be better shown by another method, by means of which they are brought into sharper contrast with their surroundings. That these bodies are identical with the terminal disks described above may fairly be assumed.

The most important question, whether the nerves extend throughout the entire odontoblastic zone, could be answered positively by Bethe's modification of the methylene-blue process. By this method nerve-colorings appear only here and there in the inner part of the pulp. At first I was afraid that the seemingly diffuse blue tint of the odontoblastic zone rendered it impossible to recognize nerve-colorings, and for this reason I discarded a whole series of specimens which had been prepared by this method. However, on a very bright day, when I examined this zone under higher magnifying power, and with the use of oil immersion, the diffuse coloring resolved itself into one that was differentiated with considerable sharpness. (Fig. 3.)

In the so-called horns of the pulp—more correctly the crest—nerve-fibrils were universally present between the elementary cells, which were but imperfectly united to form odontoblasts; these nerve-fibrils lie in close parallel lines, and send out a large number of small lateral branches between the elementary cells, which are arranged side by side in rows. The latter are colored light blue, and their cell bodies, which by the absorption of dentinogenous substance are already partially in process of dissolution, are fairly interwoven in the delicate fibres of an intercellular net-work ; these fibres show by their marked blue tint, and by the fact that they spring from the primary nerve-fibres, that they are integral constituents of the nerve system which extends throughout the pulp.

FIG. 3.

I had previously treated dentine germs from the human and animal fœtus by the aniline-blue methods of Ciaglinsky and others (Stroebe), and I had noticed that the fibrils of Weil's zone were colored blue, like axis-cylinders, but that the dentinogenous substance was likewise colored blue. I was accordingly unable to demonstrate from these preparations that these fibrils and their branches, which extend between the still disunited elementary cells, were really nerves. Where odontoblasts had been formed there quite often appeared in them, at fairly definite intervals, narrow transverse strips of a blue tint,[1] by which the odontoblasts seemed to be divided into segments. I held these fine blue strips to be dentinogenous substance, which showed itself in this form in the

[1] Entwickelungsgeschichte der Zähne, in Scheff's Handbuch der Zahnheilkunde, 1 Band, Seite 280–281.

odontoblasts. Erwin Hoehl,[1] likewise observed this phenomenon, but correctly explained these transverse strips as fibrils of the intercellular net-work. That this net-work is formed for the most part by elements of the nerve system I was now able to demonstrate conclusively by the methylene-blue and other (Nikiforoff) methods.

In this connection my method with formic acid proved peculiarly efficacious. In the odontoblasts, which are very darkly colored in their medial and central parts, one can still recognize in many places the outlines of what were originally elementary cells; between these dark fibres appear, which continue towards the centre as a fibrillary net-work of Weil's zone, and which, in many portions of the specimen, can be traced to the sharply-colored medullary nerves.

In the odontoblastic zone, on account of the dark color in the specimens, I was only occasionally able to demonstrate transverse strips or fibrils running transversely; but the intercellular net-work of Weil's zone was all the more apparent. I note here, parenthetically, that, according to my more recent investigations, Weil's zone, despite its apparent poverty in cells, nevertheless consists of a dense aggregation of cells that have been transformed by a peculiar chemical change. Some of these cells have remained unaltered, and appear in this zone as isolated nucleated cells, while the rest, having lost their nucleus, are indicated for the most part only by the finer fibres of the intercellular net-work. The fibrous constituents of this net-work developed originally between the former elementary cells (mesodermic cells), enclosing the latter as in a genuine sheath. After the transformation of the cells, during which their contents first become transparent and then granular and clouded, the net-work is at first more prominent, and impresses one as a separate zone. During the next stage of evolution, in which row after row of the transmuted cells develop dentinogenous substance and unite to form odontoblasts (conjugation of the elementary cells), the intercellular net-work is again invisible, on account of the optic properties of this dentinogenous substance, and does not appear beneath the structural outline of the dentine fibres and their ramifications, until the sections between these have been transformed by the absorption of lime salts into basic substance.

That cells which belong to the nerve system do occur between the odontoblasts is proved both by preparations made by me

---

[1] Beitrag zur Histologie der Pulpa und des Dentins, von Erwin Hoehl, Arch. f. Anat. u. Physiol., 1896, Anat. Abtheilung, Seite 38 u. 44.

according to Method 2 and also by those made according to Method 6. These cells are very narrow, on an average from two to four $\mu$ broad and from four to seven $\mu$ long; they have been overlooked heretofore by others on account of their small size and on account of the difficulty experienced in coloring them. (Fig. 3.) By Bethe's methylene-blue method they are colored a light blue, violet, or greenish-gray blue; by the osmic-acid method, dark brown or dark gray. As has been already stated in the introduction, the peculiar chemical and optical properties of the odontoblastic zone serve to explain why the morphological constituents are so difficult to recognize, both there and in the membrana præformativa. The more nearly the odontoblastic zone approaches the dental process the more thoroughly does it seem to be saturated with a hyaline substance, which opposes an almost insurmountable obstacle to all investigation.

The question whether ganglion cells occur in the pulp of the teeth could be answered by the aid of the methylene-blue method, to the extent that remarkably large cells connected with at least two nerve-fibres occur, especially in the neighborhood of the larger blood-vessels,—*i.e.*, in the axial portion of the pulp. (Fig. 4.) I

Fig. 4.

Nerves from the central part of the pulp of a sheep's tooth, treated by Method 2. Zeiss, objective D, Oc. iv.—N, nerve-fibres; GZ, ganglion cells.

could not determine the internal structure of these cells by this method. The transition of axis-cylinders from one nerve-fibre to another, the respective interchange of the axis-cylinders of different nerves, could be established in many parts of the pulp.

### SUMMARY OF RESULTS.

1. The nerves of the pulp can be distinguished, according to their location and distribution, into central and parietal nerves. The former exhibit more strongly developed stems, divide generally at about half the height of the crown into nerve-bundles, which again resolve themselves just below the odontoblastic zone into parallel lines of primary fibres. They enter the dentine principally by the so-called horns of the pulp,—more correctly the crest,—passing through the odontoblastic zone. The nerve stems of the parietal system are more slender, but much more numerous than those of the central. They spread in two different directions, an axial and a radial. The former have the same direction as the long axis of the tooth, the latter that of the dentine canals.

2. Although likewise belonging to the parietal system, its outermost stratum forms a system in itself. In the form of a thin leaf it passes around the entire dentine germ, its groups of fibres following a course between the membrana præformativa and the peripheral parts of the odontoblasts, or between mesodermic cells.

3. The more strongly developed nerve-fibres of the central system form no proper plexus in the pulp; those of the parietal system form temporarily in young pulps a plexus.

4. The medullary sheaths of the nerves of the pulp are secondary formations, which can only be determined generally and positively in teeth that have been cut. When the nerves are fused in the dentine the medullary border for the most part again disappears.

5. The odontoblastic zone is traversed in the most varied directions by nerve-fibres; these were already present as intercellular fibrils between the (mesodermic) cells of the dentine germ from which the odontoblasts are formed.

6. Many nerve-fibres have free terminations in the pulp, others show knobby enlargements, beyond which for the most part a short supplementary fibre extends; others again terminate in peculiar cell-like bodies with the form of disks; the latter occur frequently in the odontoblastic zone.

7. In the central part of the pulp large cells appear, which, by reason of their anatomical situation, must be considered as ganglion cells.

## Figures and Tables

**Fig. 1. f1:**
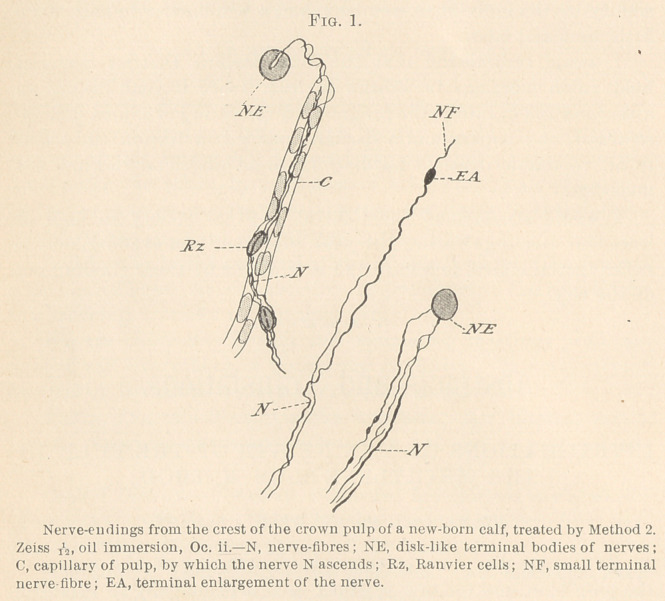


**Fig. 2. f2:**
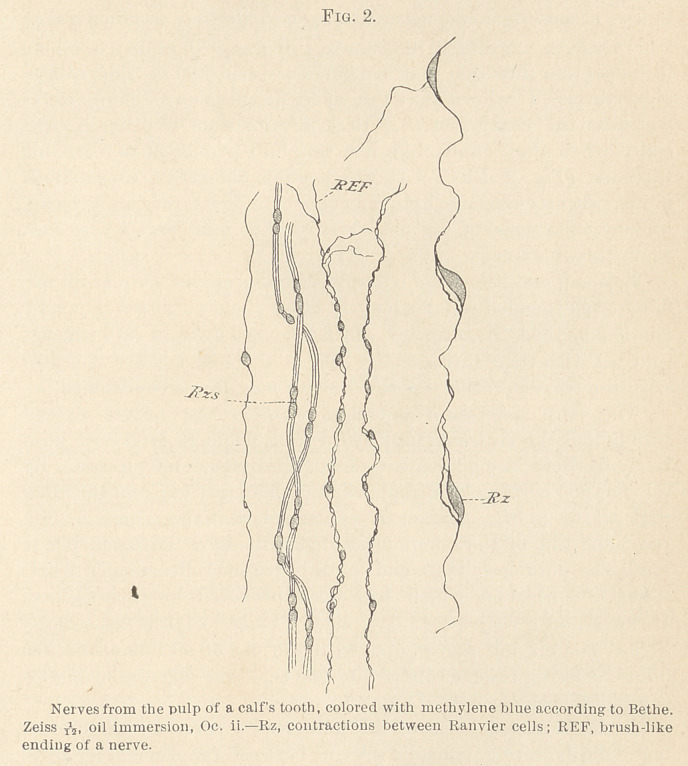


**Fig. 3. f3:**
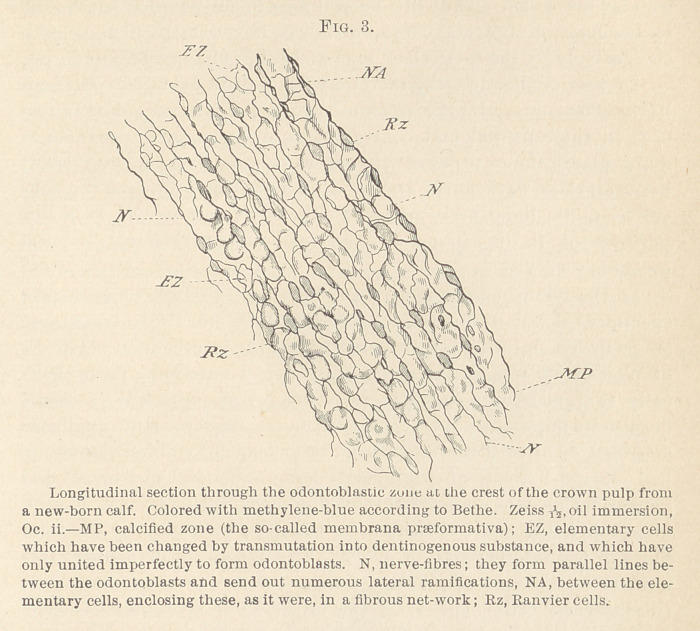


**Fig. 4. f4:**